# Synthesis and electrical characterization of intrinsic and in situ doped Si nanowires using a novel precursor

**DOI:** 10.3762/bjnano.3.65

**Published:** 2012-07-31

**Authors:** Wolfgang Molnar, Alois Lugstein, Tomasz Wojcik, Peter Pongratz, Norbert Auner, Christian Bauch, Emmerich Bertagnolli

**Affiliations:** 1 Institute of Solid State Electronics, TU-Wien, Floragasse 7, A-1040 Vienna, Austria; 2 Institute of Solid State Physics, TU-Wien, Wiedner Hauptstrasse 8/052, A-1040 Vienna, Austria; 3 Spawnt Research GmbH, Entwicklungszentrum Wolfen, Kunstseidenstrasse 6, D-06766 Bitterfeld-Wolfen; 4 Johann Wolfgang von Goethe-University, Max-von-Laue-Strasse 7, D-60438 Frankfurt am Main, Germany

**Keywords:** chemical vapour deposition, field-effect transistor, oligosilanes, radiation-induced nanostructures, silicon nanowires, vapor–liquid–solid mechanism

## Abstract

Perchlorinated polysilanes were synthesized by polymerization of tetrachlorosilane under cold plasma conditions with hydrogen as a reducing agent. Subsequent selective cleavage of the resulting polymer yielded oligochlorosilanes Si*_n_*Cl_2_*_n_*_+2_ (*n* = 2, 3) from which the octachlorotrisilane (*n* = 3, Cl_8_Si_3_, OCTS) was used as a novel precursor for the synthesis of single-crystalline Si nanowires (NW) by the well-established vapor–liquid–solid (VLS) mechanism. By adding doping agents, specifically BBr_3_ and PCl_3_, we achieved highly p- and n-type doped Si-NWs by means of atmospheric-pressure chemical vapor deposition (APCVD). These as grown NWs were investigated by means of scanning electron microscopy (SEM) and transmission electron microscopy (TEM), as well as electrical measurements of the NWs integrated in four-terminal and back-gated MOSFET modules. The intrinsic NWs appeared to be highly crystalline, with a preferred growth direction of [111] and a specific resistivity of ρ = 6 kΩ·cm. The doped NWs appeared to be [112] oriented with a specific resistivity of ρ = 198 mΩ·cm for p-type Si-NWs and ρ = 2.7 mΩ·cm for n-doped Si-NWs, revealing excellent dopant activation.

## Introduction

As potential building blocks for nanoelectronics [1–2], bio-chemical sensors [3–4], light-emitting devices with extremely low power consumption, and solar cells [5], nanotubes [6] and NWs [7] have drawn a lot of interest during the last two decades. To tune the NWs for their respective applications, their electrical and optical properties, which strongly depend on the diameter [8] as well as the crystallographic orientation [9] and defect structure [10] of the NW, must be carefully adjusted. Several synthesis techniques have proven suitable to achieve NWs with tailored properties, namely chemical vapor deposition (CVD) [11], metal–organic CVD [12], molecular-beam epitaxy [13] and laser ablation techniques [14]. In this work we focus on the well-established VLS growth mechanism [15–16], which has shown remarkable potential in the fabrication of straight, crystalline, nanometre-sized wires. During VLS growth a Si precursor is introduced, which is cracked and dissolved into the catalytic liquid phase. Generally Au is used as the catalyst on Si substrates, forming a liquid alloy with a eutectic temperature of 364 °C, which, upon supersaturation, nucleates the growth of a Si-NW.

In previous work [17] we investigated the crucial importance of substrate preparation in the case of Au-catalysed NWs grown by the VLS mechanism. Removal of silicon oxide shortly before catalyst deposition proved to be decisive for achieving epitaxy and crystallinity. The oxide on top of a Si substrate can also be removed during growth by using SiCl_4_ as a precursor. Gaseous HCl, a byproduct of SiCl_4_ decomposition in the presence of H_2_, etches the native oxide, providing a clean substrate surface for epitaxial NW growth. The same effect can be utilized by intentionally adding HCl to the growth atmosphere [18]. For such VLS grown NW dopants can be introduced either through particular catalyst particles, such as In [19], Al [20] or Ga [21], which become partly incorporated into the NW during growth and thus work as p-type dopants themselves, or by adding a small amount of dopant intentionally to the Au catalyst particle [22]. Much more common and effective is to add a gaseous dopant, such as PH_3_, B_2_H_6_ or B(CH_3_)_3_, to the precursor gas feed during growth. Thus, for example, p–i–n^+^-type doped Si-NW heterostructures with a resistivity of a few mΩ·cm have been achieved [20]. Unfortunately, such in situ doping can negatively affect the actual growth process. B_2_H_6_ for example triggers the formation of an amorphous Si shell [23], whereas PH_3_ reduces the growth rate and completely inhibits NW growth at higher PH_3_ partial pressures [24]. Furthermore, the doping often appears to be radially inhomogeneous and diameter dependent [25]. In this paper we discuss the electric properties of Si-NWs grown with Si_3_Cl_8_ [26] as well as peculiarities of the in situ doped NW synthesis using this precursor in combination with BBr_3_ or PCl_3_.

## Experimental

For the synthesis of perchlorinated polysilanes an industrial microwave device (MX 4000, Muegge Electronics GmbH), connected to a rectangular waveguide that leads into a reaction chamber, was used. The reactor itself consisted of a quartz-glass tube, inserted into the microwave cavity, with the axis of the waveguide being perpendicularly aligned to the reaction tube. Prior to use, the reaction apparatus was carefully dried by heating in vacuum. A gaseous mixture of 40 mL (59.2 g) of SiCl_4_ and 17 L of H_2_ was introduced into the reaction chamber and the pressure was carefully adjusted to 2 mbar. By powering a solid-state Tesla transformer, a glow discharge (10 W) of about 12 cm in length was generated. Then, pulsed microwave radiation was used to initiate plasma filling of the whole reaction tube at a length of 8 cm. The microwave pulse duration was set to 1 ms at 4 kW followed by a pause of 59 ms, resulting in an average power level of 67 W. The gas mixture was consumed within 200 min and a white-brown waxy solid (22 g) was deposited on the tube walls. This polymeric material was dissolved in a small amount of SiCl_4_ and isolated from the reactor. Cryoscopic investigations showed the molecular weight of the polymer to be around 1700 g/mol, which proves the formation of a perchlorinated polysilane (SiCl_2_)*_n_* and/or of Si*_n_*Cl_2_*_n_*_+2_, with an average chain length of about *n* = 17 for (SiCl_2_)*_n_* or *n* = 16 for Si*_n_*Cl_2_*_n_*_+2_. Moreover the molar ratio of Si/Cl was found to be 1:2 by titration after Mohr [27]. Similar to the process described in the literature [28], 50 g of the perchlorinated polysilane were dissolved in 500 mL of SiCl_4_ and placed in a 1 L flask equipped with a reflux condenser, a stirrer and a gas inlet. The reflux condenser was connected with a cooling trap (−20 °C). Dry chlorine gas was slowly passed through the reaction solution at the reflux temperature of SiCl_4_ (~57 °C). The reflux temperature was slowly raised but kept below the boiling point of Si_2_Cl_6_, whereupon most of the SiCl_4_ was distilled off. After 10 h the slightly yellow solution was distilled at normal pressure to separate SiCl_4_, Si_2_Cl_6_ (*T*_B_ = 145 °C/760 mmHg, 25 g), and Si_3_Cl_8_ (*T*_B_ = 215 °C/760 mmHg, 16 g). Higher oligosilanes remained in the distillation residue and were not isolated. For characterization of the precursor compounds, Si_2_Cl_6_ and Si_3_Cl_8_ were identified by their characteristic ^29^Si NMR chemical shifts (Si_2_Cl_6_, δ = −6.4 ppm; Si_3_Cl_8_, δ = −3.7 (-SiCl_3_), −7.4 ppm (-SiCl_2_-)) [29–30] and by GC–MS measurements. Trace analysis was performed by ICP–MS measurements. For the preparation of the doped samples, BBr_3_ and PCl_3_ were added to the oligosilanes in very small quantities (100 ppm). After distillation the doped oligosilanes were directly used for NW growth in an APCVD system.

The main components of the APCVD growth chamber are a horizontal tube furnace with three individually controlled heating zones, a quartz tube connected to a gas feed, and a pumping unit. To supply the furnace with gaseous OCTS precursor a saturator was utilized with He as the feed gas. A more detailed description of the growth apparatus is given in [30]. As substrates, pieces of Si (111) were cleaned with acetone, rinsed with propan-2-ol and blown dry with N_2_. The native oxide was removed by buffered hydrofluoric acid (BHF; HF/NH_4_F 7:1) resulting in a hydrogen-terminated Si surface. Au colloids (80 nm) in propan-2-ol were then dropped onto the substrate and after evaporation of the solvent and an additional dip in BHF, the samples were immediately introduced into the APCVD system. The reactor was evacuated and purged with He, three times, to remove any traces of air. Thereafter the temperature was ramped up with the samples still outside of the heated zone, under a flow of 100 sccm of He. When the furnace reached the final growth temperature the sample holder was transferred into the growth region with the aid of a magnetic specimen-transport system, enabling accurate and fast placement of the samples at desired temperatures without breaking the vacuum. It turned out that annealing of the samples for 30 min at 800 °C prior to growth improved epitaxy considerably. After this pre-annealing, OCTS was introduced into the growth atmosphere with a partial pressure of ~0.03 mbar by routing the He through the saturator. Taking into account the temperature gradient of the furnace, process temperatures from 900 to 400 °C in steps of 100 °C were investigated simultaneously within the same growth sequence, which gave the most direct and reliable information about the influence of the growth temperature [30]. After the standard growth duration of 60 min, the sample holder was pulled out of the heating zone with the magnetic specimen-transport system, enabling a very fast cool down of the samples, which still remained in the growth atmosphere. Finally, the precursor gas flow was stopped, and the quartz tube was purged with He for a further 5 min before the sample was removed from the APCVD system.

For contacting the NWs, 200 × 200 µm^2^ Au pads were structured on a highly doped Si (100) wafer, capped with 80 nm Al_2_O_3_, by photolithography and lift-off techniques. VLS-grown Si-NWs were then removed from their growth substrates by ultrasonication in propan-2-ol. Subsequently the NWs were randomly distributed by dropping the suspension onto the above mentioned Si(100) wafer with prepatterned Au pads. Finally the NWs were connected to the prepatterned Au pads by electron beam lithography, Ni sputter deposition and lift-off techniques.

## Results and Discussion

**Figure 1:**
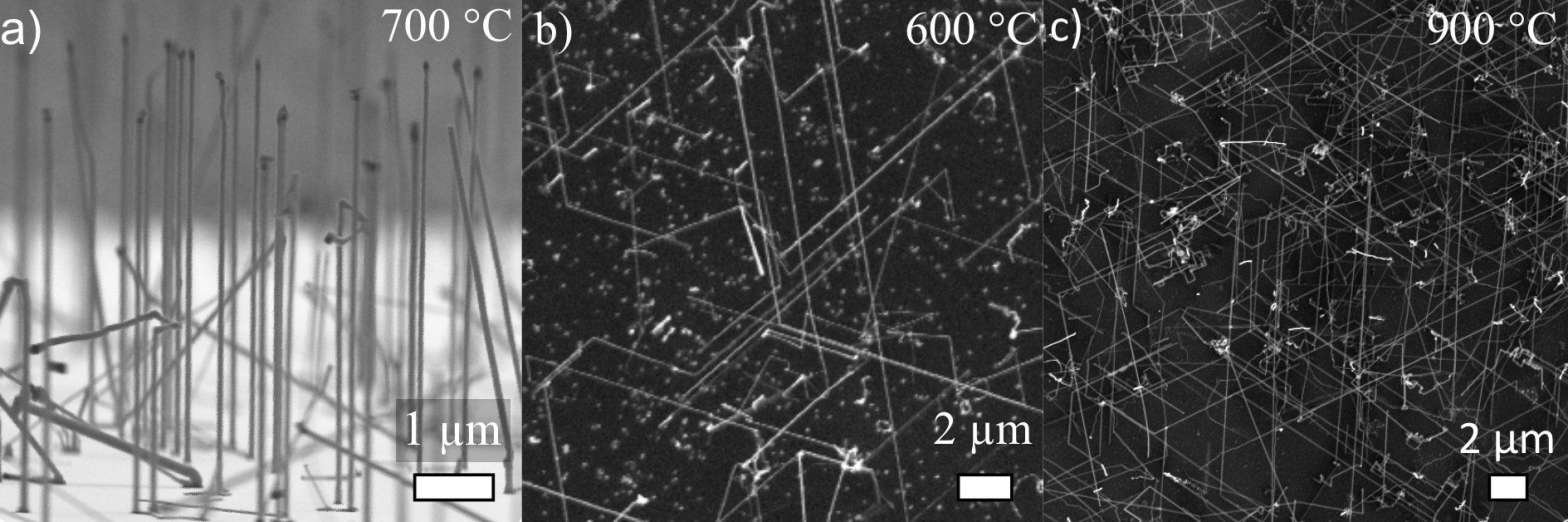
**(**a) tilted-view SEM image of Au-catalysed NWs grown at 700 °C with OCTS, (b) top-view SEM image of boron-doped Si-NWs grown at 600 °C, (c) SEM image of phosphorus-doped NWs grown at 900 °C.

Single-crystalline and epitaxial Si-NWs were grown by using OCTS as a precursor and Au colloids at a growth temperature of 700 °C, with a pre-annealing of the samples at 800 °C for 30 min. The thus synthesized NWs, shown in Figure 1a, were 4 to 10 µm long and 80 to 100 nm thick.

Based on such an optimized NW synthesis procedure, we added BBr_3_ to the OCTS precursor expecting the formation of p-type doped Si-NWs. However, the addition of BBr_3_ strongly affects the growth behaviour. Notably, effective growth of B-doped Si-NWs with OCTS and BBr_3_ requires a reduction of the growth temperature and the addition of H_2_. Remarkably, the addition of H_2_ during the growth of intrinsic NWs causes significant etching under the given experimental conditions [30]. However, adding 10 sccm of H_2_ for the synthesis of p-type doped NWs yielded epitaxial, 10 to 20 µm long and 80 to 150 nm thick Si-NWs at a growth temperature of 600 °C (Figure 1b). NWs were observed in large quantity down to temperatures of 400 °C, but epitaxy deteriorated with decreasing temperature. To achieve n-type Si-NWs, PCl_3_ was added to OCTS. Again effective NW growth required the addition of H_2_ to the growth atmosphere and a higher growth temperature of at least 800 °C. Furthermore, to achieve epitaxial NW growth, the colloids were replaced by a 2 nm thick sputter-deposited Au layer. Epitaxial NWs about 60 nm to 150 nm in diameter and up to 30 µm long are shown in Figure 1c.

**Figure 2:**
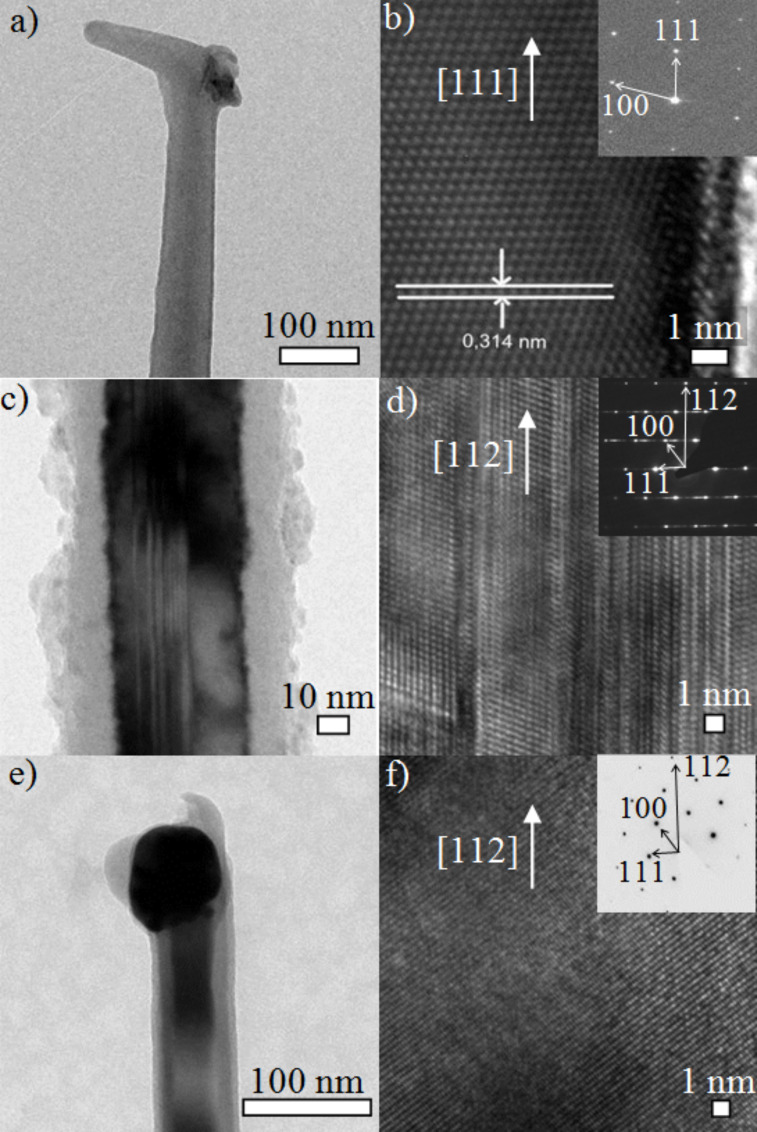
TEM images (a), (c) and (e) show impressions of intrinsic, B-doped and P-doped NWs respectively. Analogously (b), (d) and (f) represent the respective HRTEM images with diffraction images in the inset.

Summarizing the synthesis results, one has to note that even small amounts (ppm) of the doping agent change the growth behaviour considerably. For pure OCTS we achieved effective Si-NWs growth in the temperature regime from 600 to 900 °C without any H_2_, though with varying quality. With the addition of BBr_3_, NWs growth was restricted to the temperature regime between 400 and 600 °C, although this required the addition of H_2_ to the growth atmosphere. Briand et al. [31] also reported lower growth temperatures when adding B_2_H_6_ to SiH_4_, as boron promotes the decomposition of the precursor and therefore increases the growth rate. With PCl_3_ as the dopant, at least 800 °C, 20 sccm H_2_, and a 2 nm layer of Au were needed to produce epitaxial NWs in considerable quantity. For a more detailed view of the morphology of the intrinsic and doped Si-NWs we performed HRTEM investigations. The TEM image in Figure 2a shows a slightly tapered, intrinsic Si-NW with a catalytic particle atop. The HRTEM micrograph of the crystalline core in Figure 2b shows clearly the Si(111) atomic planes (separation 3.14 Å) perpendicular to the NW axis. The reciprocal lattice peaks in the diffraction pattern (inset in Figure 2b) prove that the growth axis is [111], and previous work on Si-NWs grown with SiH_4_ revealed, vertical {112} facets [32]. The NWs are usually free of dislocations and stacking faults and are covered by a very thin oxide layer.

As already mentioned above, the addition of B_2_H_6_ requires the modification of growth parameters, such as temperature and feed-gas composition, to achieve effective NW growth. Moreover the addition of the dopant species, and thus the expected insertion of B into the lattice of the Si-NW, also influences the morphology and crystal orientation. Nevertheless, they have comparable diameters to those of the intrinsic NWs grown with pure OCTS. The growth orientation of the p-type doped NWs appears to be [112], as shown in the HRTEM image and the respective diffraction pattern (Figure 2c,d). Stacking faults run along the entire NW from the base to the top, and the crystalline core is enwrapped by an amorphous shell. A similar amorphous shell was also observed by Lauhon et al. [33] on addition of B_2_H_6_ to the growth atmosphere with the SiH_4_ precursor. The images in Figure 2e and Figure 2f show that also PCl_3_ affects NW growth by changing the growth direction. Such epitaxial NWs grow preferentially along the [112] direction, like their B-doped counterparts. The NWs themselves are rod-like, exhibit good crystallinity, and feature no observable defects or stacking faults.

**Figure 3:**
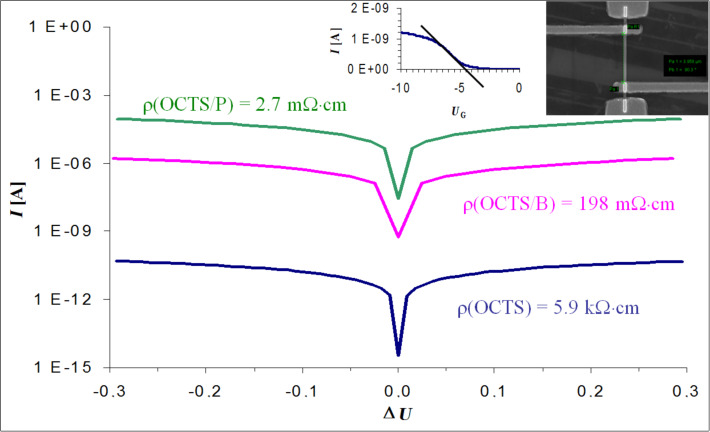
Semilogarithmic *I*/*V* plot of intrinsic, p- and n-type NWs. The calculated specific resistivity values are shown next to the respective curves. The transfer characteristic of the intrinsic NW integrated into a back-gated Schottky-barrier NW-FET and a SEM image of a four-point setup is shown in the inset.

To test the activation of the dopants in the NWs, electrical characterization was performed with back-gated Schottky-barrier NW-FETs and four-point measurement modules. The results of the four-point measurements and the back-gated measurements are illustrated in Figure 3. The four-point measurements of nominally intrinsic NWs grown with pure OCTS revealed a resistivity of about 5.9 kΩ·cm. This is in accordance with the results of Heath et al. [34], who reported a specific resistivity of intrinsic NWs, grown with SiH_4_ as a precursor, of about 1 kΩ·cm. Back-gated measurements revealed an unintentionally p-type doping leading to a threshold voltage of −4.5 V (Figure 3 inset). Such p-type behaviour is observed for most intrinsic VLS-grown Si-NWs and can be attributed to hole accumulation at the surface due to trapped negative surface charge, although contributions from impurities such as Au and O cannot be excluded completely [35].

For the as grown intentionally p-doped NWs, we determined a resistivity of about 862 Ω·cm. This rather high resistivity arose from the immense amorphous shell (see Figure 2c) wrapped around a highly crystalline core. Thus, in the case of the intentionally B-doped NWs, an annealing step at 470 °C for 2 min was required to achieve reliable contacts. Subsequent measurements revealed a specific resistivity of 198 mΩ·cm (Figure 3), which corresponds to an active dopant concentration of 10^17^ cm^−3^ in bulk Si. A similar behaviour was also reported by Lauhon et al. [35]. The p-type doped NWs showed a high resistivity in the kΩ·cm regime, which can be reduced upon annealing to a few mΩ·cm as a result of complete crystallisation. Adding PCl_3_ to the growth atmosphere results in n-type Si-NWs with a resistivity of 2.7 mΩ·cm, corresponding to an active P concentration of 3 × 10^19^ cm^−3^ in bulk Si. Remarkably this is more than six orders of magnitude lower than the resistivity of the intrinsic NWs in this work. Due to the high doping level we observed no channel modulation in response to the gate voltage for the doped NWs integrated in back-gated FETs.

## Conclusion

In conclusion, OCTS appeared to be a favourable precursor for VLS synthesis of intrinsic as well as in situ doped NWs. However, the addition of BBr_3_ and PCl_3_ as doping agents requires a careful tuning of the growth parameters. NWs synthesized with pure OCTS exhibit a growth orientation of [111], while the doped NWs appear to be predominantly [112] oriented. Finally the electrical characterisation revealed a resistivity of 5.9 kΩ·cm for intrinsic Si-NWs, which appeared to be unintentionally p-type doped and 198 mΩ·cm and 2.7 mΩ·cm for the B- and P-doped NWs, respectively. This proves that the electronic properties of Si-NWs grown with OCTS as Si precursor can be tuned according to the desired applications. Also the growth orientation can be controlled, which may prove useful for device integration. Therefore OCTS-grown NWs represent promising new alternatives in the upcoming fields of nanoelectronics, optics, thermoelectronics and sensor devices [36].

## References

[1] Duan X, Huang Y, Lieber C M (2002). Nano Lett.

[2] Javey A, Nam S, Friedman R S, Yan H, Lieber C M (2007). Nano Lett.

[3] Cui Y, Lieber C M (2001). Science.

[4] Zheng G, Patolsky F, Cui Y, Wang W U, Lieber C M (2005). Nat Biotechnol.

[5] Pettersson H, Trägårdh J, Persson A I, Landin L, Hessman D, Samuelson L (2006). Nano Lett.

[6] Martel R, Derycke V, Lavoie C, Appenzeller J, Chan K K, Tersoff J, Avouris P (2001). Phys Rev Lett.

[7] Duan X, Huang Y, Cui Y, Wang J, Lieber C M (2001). Nature.

[8] Brus L (1994). J Phys Chem.

[9] Yorikawa H, Uchida H, Muramatsu S (1996). J Appl Phys.

[10] Mozos J L, Machado E, Hernandez E, Ordejon P (2005). Int J Nanotechnol.

[11] Wagner R S, Ellis W C (1964). Appl Phys Lett.

[12] Arakawa Y (1994). Solid-State Electron.

[13] Martelli F, Piccin M, Bais G, Jabeen F, Ambrosini S, Rubini S, Franciosi A (2007). Nanotechnology.

[14] Wang N, Tang Y H, Zhang Y F, Lee C S, Lee S T (1998). Phys Rev B.

[15] Levitt A P (1970). Whisker Technology.

[16] Givargizov E I (1975). J Cryst Growth.

[17] Lugstein A, Hyun Y J, Steinmair M, Dielacher B, Hauer G, Bertagnolli E (2008). Nanotechnology.

[18] Sharma S, Kamins T I, Stanley Williams R (2004). J Cryst Growth.

[19] Iacopi F, Vereecken P M, Schaekers M, Caymax M, Moelans N, Blanpain B, Richard O, Detavernier C, Griffiths H (2007). Nanotechnology.

[20] Björk M T, Knoch J, Schmidt H, Riel H, Riess W (2008). Appl Phys Lett.

[21] Sharma S, Sunkara M K (2004). Nanotechnology.

[22] Schmidt V, Riel H, Senz S, Karg S, Riess W, Gösele U (2006). Small.

[23] Lew K-K, Pan L, Bogart T E, Dilts S M, Dickey E C, Redwing J M, Wang Y, Cabassi M, Mayer T S, Novak S W (2004). Appl Phys Lett.

[24] Schmid H, Björk M T, Knoch J, Karg S, Riel H, Riess W (2009). Nano Lett.

[25] Xie P, Hu Y, Fang Y, Huang J, Lieber C M (2009). Proc Natl Acad Sci U S A.

[26] Molnar W, Lugstein A, Pongratz P, Auner N, Bauch C, Bertagnolli E (2010). Nano Lett.

[27] Jander G, Blasius E (1995). Einführung in das anorganisch-chemische Praktikum.

[28] Schmeisser M, Voss P (1964). Z Anorg Allg Chem.

[29] Sharp K G, Sutor P A, Williams E A, Cargioli J D, Farrar T C, Ishibitsu K (1976). J Am Chem Soc.

[30] Marsmann H C, Raml W, Hengge E (1980). Z Naturforsch.

[31] Briand D, Sarret M, Kis-Sion K, Mohammed-Brahim T, Duverneuil P (1999). Semicond Sci Technol.

[32] Lugstein A, Andrews A M, Steinmair M, Hyun Y-J, Bertagnolli E, Weil M, Pongratz P, Schramböck M, Roch T, Strasser G (2007). Nanotechnology.

[33] Lauhon L J, Gudiksen M S, Wang D, Lieber C M (2002). Nature.

[34] Yu J-Y, Chung S-W, Heath J R (2000). J Phys Chem B.

[35] Zhang S, Hemesath E R, Perea D E, Wijaya E, Lensch-Falk J L, Lauhon L J (2009). Nano Lett.

[36] Sarkar J, Khan G G, Basumallick A (2007). Bull Mater Sci.

